# A hidden mismatch between experiences of young athletes with overuse injuries of the wrist and sports physicians’ perceptions: a focus group study

**DOI:** 10.1186/s12891-019-2616-y

**Published:** 2019-05-20

**Authors:** Laura S. Kox, Jip Opperman, P. Paul F. M. Kuijer, Gino M. M. J. Kerkhoffs, Mario Maas, Monique H. W. Frings-Dresen

**Affiliations:** 1 Department of Radiology and Nuclear Medicine, Amsterdam UMC, University of Amsterdam, Meibergdreef 9, 1105 AZ Amsterdam, The Netherlands; 2 Coronel Institute of Occupational Health, Amsterdam Public Health research institute, Amsterdam UMC, University of Amsterdam, Meibergdreef 9, 1105 AZ Amsterdam, The Netherlands; 3 Department of Orthopedic Surgery, Amsterdam UMC, University of Amsterdam, Meibergdreef 9, 1105 AZ Amsterdam, The Netherlands; 4 Academic Center for Evidence-based Sports medicine (ACES), Meibergdreef 9, 1105 AZ Amsterdam, The Netherlands; 5 Amsterdam Collaboration for Health and Safety in Sports (ACHSS), International Olympic Committee (IOC) Research Center AMC/VUmc, Meibergdreef 9, 1105 AZ Amsterdam, The Netherlands

**Keywords:** Sports injury, Qualitative research, Wrist, Paediatrics, Outcome measures, Pain, Focus group, Young athlete, Early detection

## Abstract

**Background:**

Although overuse wrist injuries can have serious consequences, young athletes often do not immediately report their injury to a physician. This qualitative study aimed to identify symptoms and limitations related to overuse wrist injuries that young athletes consider important and to compare those with sports physicians’ opinions, in order to improve the diagnostic process for early identification of overuse wrist injuries.

**Methods:**

Twenty-one athletes aged 13–25 years in wrist-loading sports (gymnastics, tennis, judo, field hockey, volleyball and rowing) with a (previous) overuse wrist injury were included. In five focus groups, participants discussed important signals and limitations of their injury, as well as a list of relevant items previously composed by sports physicians. Data were grouped into themes and (sub)categories and subsequently coded.

**Results:**

Of the resulting 224 signals and 80 limitations, respectively 81 and 20 were labelled important. Athletes considered both pain and limitations during daily life activities important indicators of overuse wrist injury, as well as long pain duration, acute onset of pain, and accompanying symptoms like swelling, cracking and discoloration. All of the sports physicians’ items were also considered important by the athletes, but sport-related pain and limitations were regarded by many athletes as a natural part of their sport.

**Conclusions:**

Discrepancies exist between the opinions of young athletes and sports physicians on sport-related pain reporting and competing regardless of pain or limitations. Although clinicians may be inclined to focus on these aspects, they are advised to also inquire specifically about limitations and pain during daily life activities in young athletes with overuse wrist injuries.

**Electronic supplementary material:**

The online version of this article (10.1186/s12891-019-2616-y) contains supplementary material, which is available to authorized users.

## Background

Awareness of overuse injuries among young athletes is increasing. Early sport specialization and increasing training hours have been shown to contribute to higher rates of overuse injuries in young athletes [1]. In numerous youth sports, for example gymnastics and rowing, overuse wrist injuries are a well-known problem, with prevalence rates for wrist pain of 32–73% [2]. In a Dutch cohort of young tennis players (ages 11–14 years), 38% of all upper extremity injuries were overuse wrist injuries. [3] Previously, six Olympic youth sports have been described, among which gymnastics and tennis, which involve frequent loading of the wrist and are popular in the Netherlands [2, 4]. Wrist injuries and their consequences are particularly relevant for the young athletic population in view of possible functional impairments later in life.

Overuse wrist injury mechanisms differ per sport, including carpal stress fractures and tendon injuries in tennis and rowing, repetitive sprains in volleyball, and physeal stress injury in gymnastics. [5–8] The long-term consequences of overuse wrist injuries include degenerative conditions that often cause pain and functional limitations [9]. In the more immediate stage of many overuse injuries early diagnosis can promote quicker care and recovery and thus faster return to play. Less time lost to injury can be very important in maintaining an athlete’s quality of life [10]. Although the importance of early diagnosis of such injuries is obvious to clinicians, young athletes often do not report overuse wrist injuries to a physician immediately [8]. Young gymnasts often do not even report painful incidents to the wrist to their coaches even if they occur frequently [11].

Studies of young athletes have shown that many view competing or training while in pain as normal [12, 13]. This idea that injury in sports is normal and that playing while in pain is accepted, by athletes as well as by coaches, is described as the “risk-pain-injury paradox” [14]. Particularly young elite or highly perfectionistic athletes have shown a high degree of acceptance of health risks related to their sport [15]. A study among young gymnasts showed that they were able to identify when to stop when they experienced an acute injury, but that this was much less likely when chronic pain was present [16]. More insight in alarming symptoms would enable the athletes themselves to recognize a possible injury and seek appropriate care sooner, to prevent serious impairments. Furthermore, when athletes do seek help, the sports physician needs to know what questions are essential to identify overuse wrist injury.

In an earlier study on this topic, sports physicians were asked what signs of overuse wrist injury they considered relevant when assessing young athletes, resulting in 61 signals, such as symptoms, and limitations that can be addressed during patient history taking [4]. To provide clinicians with an optimal yet concise set of questions to timely identify overuse wrist injuries, the perceptions of young athletes themselves are indispensable. The purpose of this qualitative study is, therefore, to identify signals (defined as any indication that injury may be present) and limitations related to overuse injury of the wrist that young athletes consider important, and to compare these items with those previously selected by sports physicians. We hypothesized that young athletes who experienced overuse wrist injuries themselves would report more specific signals and limitations related to these injuries than sports physicians, which could in the future help clinicians to identify overuse wrist injuries in a more timely manner.

## Methods

### Design

A qualitative study design using focus groups was applied in order to acquire as many signals and limitations as possible. We used the Consolidated Criteria for Reporting Qualitative Research (COREQ) checklist for reporting focus group studies (Additional file 2) [17]. The study was performed according to the Declaration of Helsinki and the Medical Ethics Review Committee of the Academic Medical Center decided that it is not subject to the Dutch Medical Research Involving Human Subjects Act (reference no. W15_142#15.0170).

### Participants

By way of purposive sampling, invitations to participate were sent to national sports federations, inviting paediatric and collegiate athletes aged 12 to 25 years with a current or previous overuse wrist injury in six popular Dutch wrist-loading sports: gymnastics, field hockey, tennis, judo, volleyball and rowing, in accordance to two previous studies [2, 4]. Each focus group contained a sample of athletes in at least three different sports, in order to encourage discussion among these athletes about potential differences and similarities in perceptions on overuse wrist injuries. The age range was chosen to achieve adequate variation in the population sample, including children as well as adolescents as their perceptions on sports performance and injury would likely differ. Fifty-one athletes volunteered and were contacted by e-mail to inform them further about the study’s purpose and methods. One of the researchers (LK) explained the study’s intentions within her research project on signalling overuse wrist injuries in young athletes. All participants and their parents or guardians (for athletes younger than 18 years) provided written informed consent to participate before the meeting.

### Setting

The meeting was supervised by one moderator, a female medical doctor and PhD (LK) currently working as a full-time researcher, with substantial experience in supervising focus group meetings and who was trained for this task by two researchers (MFD and PK). Two assistant moderators, a female PhD and medical doctor in training (CN), and a female medical student (JO), were present to take notes and ask additional questions.

Data collection took place in five meetings of approximately two hours at the Amsterdam UMC, University of Amsterdam (location AMC), in January through June 2016. During the fifth focus group interview no new information was derived from the interview, indicating that data saturation was reached. Participant numbers per focus group varied from three to five. Participants were offered drinks, snacks, travel expenses reimbursement and a gift certificate. The moderator (LK) and at least one of the other three researchers (JO, CN and EC) were present during each meeting.

### Data collection

To allow comparison with focus group results from sports physicians, the setting, types of included sports, data collection and data analysis were largely identical to this previous study [4]. The research group composed a meeting guide beforehand, based on the two main study questions, which were similar to those in the previous sports physicians’ study (Table 1) [4]. After discussion of relevant items proposed by participants, items proposed earlier by sports physicians were discussed [4]. Participants also answered a short questionnaire on demographic and training characteristics. Audio recordings were made, for which participants’ written permission had been obtained earlier. The assistant moderator took notes and wrote all relevant signals on a whiteboard.

**Table 1:** Focus group meeting guide

Main study questions	
1. What are the most important signals of overuse wrist injury for young athletes in wrist-loading focus sports?	
2. What are the most important limitations for young athletes in wrist-loading focus sports due to overuse wrist injury?	
During meeting (approximate duration 2 h)	
*Participants discuss their preferred answers to questions 1–4, with* ca. *30 min of discussion per question.*	
1. Can you tell us about your overuse wrist injury?	
2. What were reasons for you to consider this a serious injury?	
3. Which of the signals and limitations that were mentioned today, do you consider important?	
4. Take a look at the list of signals and limitations related to overuse wrist injury [list composed by focus group of sports physicians]. Which of these signals and limitations would you consider a sign of serious overuse wrist injury?	
*The items are numbered on a white board, and participants are asked to post the numbers of the signals they considered important on separate sticky notes onto the board. The resulting collection of items of perceived importance are further discussed.*	
*Prompts that can be used upon the answers of participants to questions 3 2–54:*	
a. Does this apply to everybody?	
b. Does anybody want to add something?	

At the end of each meeting, participants filled out an anonymous questionnaire rating the session’s setup and content on a 10-point scale (0= “not satisfied at all”; 10 = “very satisfied”).

### Data analysis

The audio recordings were transcribed verbatim by two researchers (LK, EC). The transcript was corrected and approved by all participants. One author (LK) coded the transcript using specialized software (MAXQDA, V12, Udo Kuckartz, Berlin). The coded transcript was checked by another researcher (PK) and coding discrepancies were discussed until 100% agreement was achieved. An inductive approach was used to organize coded items into themes derived from the data, based on the study’s research questions (Table 1). A framework on overuse wrist injuries in young athletes, derived from sports physicians, was used as guideline [4].

## Results

Forty-four athletes agreed to take part and 21 eventually participated based on availability (Table 2). Participants trained on average 12 h per week and their mean age was 18 years, with all ages between 13 and 25 years represented by at least one athlete. Their overuse wrist injuries varied from bony injuries (e.g. growth plate stress injury), to tendonitis and triangular fibrocartilage complex (TFCC) injury. All participants indicated they were sufficiently able to share their experiences and ideas and that they were satisfied with the meeting’s content and structure, rating it on average with 8.5 points out of 10.

**Table 2:** Participant characteristics

	Gymnastics	Tennis	Field hockey	Judo	Volleyball	Rowing	Total
Sex							
Female	3	2	3	2	3	2	14 ^a^
Male	0	1	1	4	0	1	7
Age (range in years)	13–21	17–20	13–22	13–25	15–19	20–24	13–25
Level							
International	0	0	1	3	0	0	4
National	2	2	1	1	1	3	10 ^a^
Regional	1	1	2	2	2	0	8 ^a^
Training hours/week (range)	7–31	4–24	4–14	3–20	2–22	12–15	4–31
Competitive events/month	1–2	4–8	4	0–4	4–8	1–2	1–8
Overuse wrist injury ^b^							
Present at moment of focus group	3	3	3	6	3	2	20
Prior to focus group participation	1	1	1	3	1	2	9

^a^ One female participant performed both gymnastics and field hockey

^b^ Some athletes reported both a present overuse wrist injury and an overuse wrist injury in the past

**Fig. 1:**
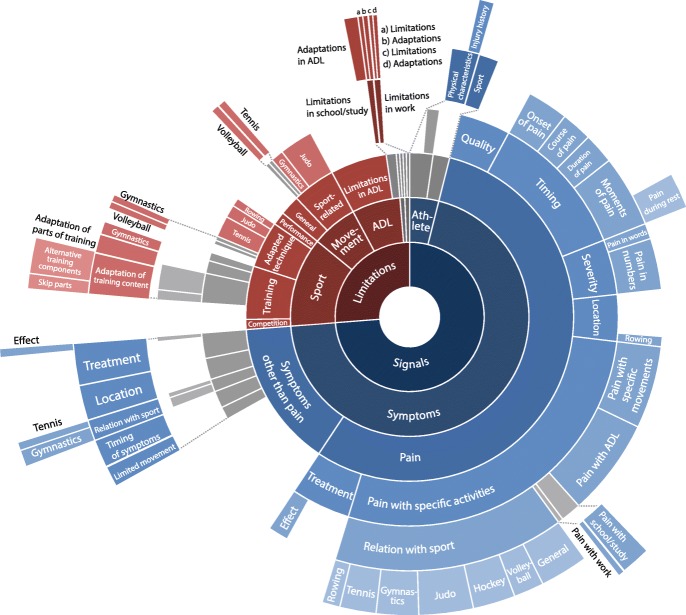
Coding system for signals and limitations related to overuse wrist injury in young athletes. Legend: The coloured inner circles represent the themes and (sub)categories of the coding system, blue indicating signals, and red indicating limitations. Very small subcategories are enlarged to display the subcategory label

**Fig. 2:**
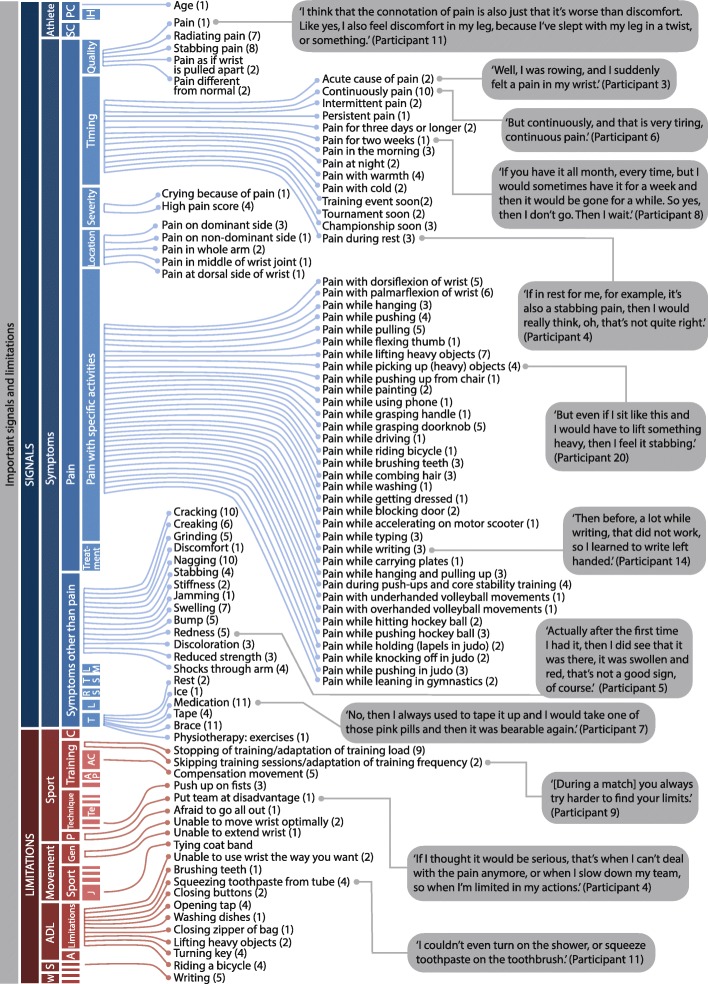
Items relating to overuse wrist injury marked as important by young athletes. Legend: Items are organized by theme and (sub)category according to the coding system shown in Fig. 1. The number (x) behind each items represents the number of athletes that considered the item important. For various items, quotes from participants are provided. Abbreviations: ADL, activities of daily life; S, school; W, work; PC, physical characteristics; SC, sports characteristics; C, competition; P, performance; Gen, general; A, adaptations; IH, injury history; LM, limited movement; TS, timing of symptoms; RS, relation with sport; L, location; T, treatment; AC, adaptation of training content; AP, adaptations of parts of training; Te, adapted technique in tennis; J, judo

The resulting items were organized into themes (224 signals and 80 limitations) and (sub)categories (Fig. 1, Additional file 1). Ninety-nine signals and 24 limitations were mentioned during the meeting, and 125 signals and 56 limitations were identified during coding of the transcripts. Eighty-one signals and 20 limitations were marked as important by participants (Fig. 2). All of the 20 items that sports physicians considered important in relation to overuse wrist injuries were considered relevant by athletes [4]. Nineteen additional items from the sports physicians’ item collection were also labelled important by athletes, including additional sport-related limitations (e.g. “change in training load”, “change in technique”) and symptoms (e.g. “reduced strength”, “stiffness”, “discoloration”).

### Signals: pain

The 224 signals were divided into categories (athlete characteristics and symptoms) and three levels of subcategories (Fig. 1). The subcategory “pain” was the largest (169 items). Multiple descriptive items relating to pain were considered important besides pain itself: “radiating pain”, “stabbing pain”, “pain as if wrist is pulled apart”, and “pain different from normal”. A participant explained why pain could be a good indication of overuse wrist injury:

*“I think that the connotation of pain is also just that it is worse than discomfort. Like yes, I also feel discomfort in my leg, because I have slept with my leg in a twist, or something.”* (Participant 11)

“Acute cause of pain” was marked as important by participants who recalled a specific day or event when first experiencing pain caused by their overuse wrist injury. This moment also played an important role in the urge to seek help. When wrist pain arose right before a training event, tournament, or championship, many participants indicated to seek help sooner. Other aspects of the timing of pain, such as pain at night or in the morning, with heat or cold, or pain in rest, were also considered worrisome and a reason to seek help.

The athletes considered both continuous and discontinuous presence of pain relevant in overuse wrist injuries. “Persistence of pain” was a tell-tale sign as well. Some participants indicated they sought help when their pain continued for three days or longer. However, others stated to postpone seeking help until pain was present for at least two weeks or even longer, especially when the pain was not continuous.

*“If you have it all month, every time, but I would sometimes have it for a week and then it would be gone for a while. So yes, then I do not go. Then I wait.”* (Participant 8)

**Fig. 3:**
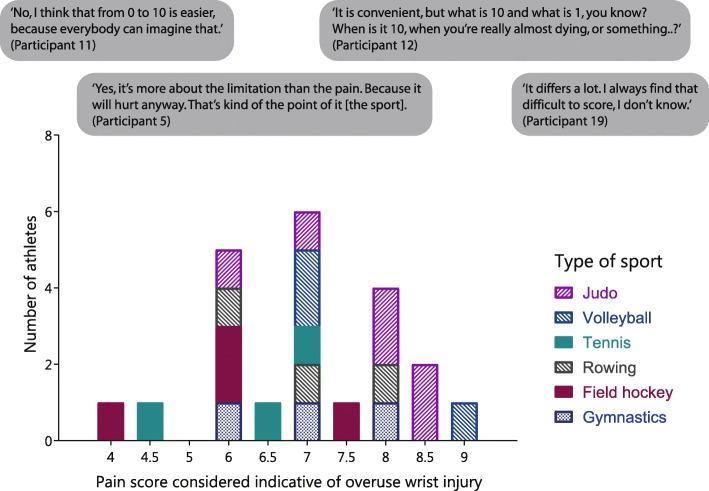
Pain scores viewed as sign of overuse wrist injury by focus group participants

In the category “severity of pain”, participants mentioned specific pain scores that they viewed as indicative of overuse wrist injury, which varied widely between 4 and 9 out of 10 (Fig. 3). Thresholds of 8 and higher were seen mainly in judokas and volleyball players, whereas tennis players and field hockey players chose thresholds of 7.5 or lower, and rowers and gymnasts found pain scores between 6 and 8 acceptable. However, none of these pain scores were considered important signs to identify a serious overuse wrist injury. Instead, “high pain score” and “crying because of pain” were preferred indicators of pain severity. Several participants mentioned a visual analogue scale (VAS) as a means for sports physicians and physiotherapists to evaluate severity of pain, some finding it difficult to use and others preferring it.

Athletes indicated to have difficulty pinpointing specific movements or locations in the wrist where pain would be reason for concern, as well as specific pain-provoking movements during sport. Most participants preferred generic descriptions of locations or wrist movements, or labelled numerous items as important because they all seemed relevant. One participant suggested inquiring instead about the severity of pain when the athlete is using the wrist to the fullest.

*“I would say, at the moment of ultimate loading [of the wrist], how much does it hurt? You yourself really know, of course, when it hurts most. You see, when you cannot brush your teeth anymore, you think ‘[expletive], I cannot do it!’ But if you do something full-on, then you really know that it will hurt a bit more. And then you can more easily indicate your pain.”* (Participant 7)

In contrast, the discussion of pain during specific activities generated numerous specific items: athletes shared extensive examples of moments when they experienced pain that made them decide to seek help. This included 18 school and work-related activities (e.g. writing, typing, carrying plates).

Participants indicated to often try various types of self-treatment before seeking medical help for overuse wrist symptoms, such as taking a period of rest, or using pain medication, tape or a brace.

### Signals other than pain

Thirteen symptoms other than pain were mentioned, including specific feelings other than pain (e.g. “nagging”, “discomfort”), other sensations inside the wrist (“clicking”, “cracking”, “creaking”, “grinding”) and visible symptoms (e.g. swelling, redness, discoloration, stiffness, reduced strength). Symptoms that can be reliably assessed by others were often considered to be a reason to seek help.

### Limitations

The 80 limitations were divided into five categories (limitations in movement, sport, daily life activities, school, and work) with three levels of subcategories (Fig. 1). Although fewer specific items were collected, some participants emphasized that limitations indicated a more serious overuse wrist injury than symptoms, such as pain (Fig. 3).

*“Well, as such, if I can make a movement, but if it hurts, […] then I take a painkiller and then I can still do it, so then I just do it.”* (Participant 4)

*“So it does hurt, but then I just power through, because it is not as if I just cannot do it at all anymore.”* (Participant 10)

If athletes experienced limitations in their sport, most were of a general nature and sport-specific limitations were mentioned only for judo. Training-related limitations like “stopping during training” and “skipping training sessions” were considered important, in contrast to competition-related items.

Limitations related to performance (“put team at disadvantage”, “afraid to go all the way”) were considered to be a reason to seek help because the injury might be serious. With respect to limitations in activities of daily living and school or study, eleven items were marked as important (e.g. “brushing teeth”, “combing hair”, “writing”). Many participants considered limitations in daily activities more important than limitations in sport, because they could often still continue their sport despite certain limitations.

## Discussion

### Indicators of overuse wrist injury

In this study young athletes provided 81 important signals and 20 important limitations that they experienced because of overuse wrist injuries. The athletes considered the items proposed by sports physicians important as well, but many viewed sport-related pain and limitations as a natural part of their sport, while they perceived pain and limitations in daily life activities as more important.

Athletes did not necessarily regard pain as indicative of overuse wrist injury. This is consistent with previous research showing that many young athletes view (minor) pain as normal, and that pain during sports performance is accepted easily. While in a study among 7542 high-school adolescents pain intensity measured by a VAS as well as duration and frequency of pain were found to be reasons to consult a physician [18], we found longer pain duration and continuous presence of pain to be more likely reasons for young athletes to seek medical help than pain measured using a VAS. Risky behaviour, including ignoring pain, was found to be common in young tennis players, and one out of every eight players aged 11–14 years reported playing with pain [3, 19]. In gymnasts aged 5–16 years, over 50% reported wrist pain during the previous 6 months, while only one third stated that pain interfered with their training [20]. Competing and training in spite of pain has been recognized as a common obstacle for accurately monitoring overuse injuries, and more focus on the consequential limitations in sports participation and performance in relation to full function has been suggested [21].

The athletes named many daily life activities that could cause pain, as well as numerous functional limitations in daily life, school, and work as important indicators of overuse wrist injury. This contrasts with statements by sports physicians discussing the same topic, who considered symptoms like pain more important than functional limitations [4]. Other studies have shown similar differences between young athletes and those around them. In one study all participating intercollegiate athletes described ways in which their injury affected their daily life activities, whereas only a third of coaches discussed the injury’s impact on an athlete’s daily life [22]. Incapacitation with respect to everyday tasks has also been identified as a major stressor in injured athletes by several other studies [23–25]. Our participants mentioned limitations in sport participation or in performance to a lesser extent than interference with everyday activities. Similarly, a study among Dutch talented young athletes (mean age, 16 years) found a fortnightly overuse injury prevalence of 12%, while athletes reported less than half of these injuries as having affected training volume or performance [26].

Several of our participants had an overuse wrist injury that first became apparent in an acute setting they remembered vividly. In studies on injury reporting, overuse is often defined as an injury that is not linked to a single identifiable event [27] and it has been suggested that overuse injuries present either gradually or acutely, after a period of chronic overload [21]. The previously mentioned focus group of sports physicians indicated that this applies to overuse wrist injuries as well [4].

### Strengths and weaknesses

We used the COREQ checklist to ensure thorough reporting [17]. The study design included multiple focus groups in order to acquire as many items as possible, and data saturation was reached after the fifth meeting. We further attempted to reduce risk of bias by making sure athletes of various ages, from various wrist-loading sports, and with different types of overuse wrist injuries and training hours were represented.

### Signals

The present findings show that young athletes’ perceptions of certain aspects of pain in relation to overuse and sports performance differ from sports physicians’ expectations. This discrepancy is, at least in part, caused by the athletes’ lack of experience with injuries at a young age. The previous sports physicians’ focus group viewed age and previous wrist injury as important signals of overuse wrist injury [4]. However, young athletes may have more difficulty discerning normal sensations of exertion from a (beginning) overuse injury than experienced adult athletes. The extensively researched concept of pain normalization and risk-taking behaviour by athletes may also be of influence [12, 28, 29]. Pain is thus often considered “part of the game”, especially by young athletes with a high drive to compete, as illustrated by studies among young gymnasts and softball players [30, 31]. Additionally, use of pain medication was considered regular practice by many participants. These factors, as well as the numerous daily activities indicated to cause pain, suggest that evaluation of pain severity using a VAS exclusively is helpful but does not provide sufficient information about the presence of an overuse wrist injury and its impact on the athlete’s life.

Symptoms other than pain that can be reliably assessed by others were also mentioned as important signals. In overuse injury monitoring, more focus on “any physical complaint” without specification has been emphasized, to reduce underestimation of overuse injuries [21]. Young athletes often experience external pressure that may keep them from reporting pain if they have no other – visible – symptoms. German elite athletes aged 14–18 years who attached great importance to their athletic identity and experienced pressure from their social environment were more willing to take physical risks (e.g. training or competing regardless of injuries) [15]. Such social pressure from coaches and parents has also been connected to overuse injuries [32, 33].

Social pressure and pain normalization may also explain why many participants regarded an identifiable acute cause of the injury as important. Although attention to acute-onset overuse injuries is increasing, many researchers still retain an overuse definition of gradual onset or absence of an acute, identifiable event. In light of the finding that pain is often considered normal by young athletes, an overuse injury may be present for some time before being acknowledged by the athlete after an acute moment of exacerbation.

### Limitations in activities

The athletes found more limitations important than the sports physicians, in particular those regarding daily activities [4]. Many considered limitations more relevant than pain when describing their overuse wrist injury. Limitations in daily life activities normally taken for granted, may indeed have more impact on athletes’ lives than limitations in sport [34]. Impairment and disability have been suggested as relevant items besides pain for future overuse questionnaires [21]. The present results show that limitations in daily life can promote young athletes’ care-seeking behaviour and may therefore be indicative of overuse wrist injury in this population.

Additionally, young athletes’ attitudes towards pain as a normal aspect of sport may influence their perception of sport-related limitations. In the literature on overuse injuries, focus lies mainly on limitations in sport, such as inability to participate. More authors are suggesting a shift from this time-loss-to-injury definition to a functional definition, although a literature review found that 29 of 30 studies still used the former [21, 35]. Three of the four questions of a recently developed overuse questionnaire refer to limitations in performance, training and competition [36]. Yet “decreased performance” was not labelled important by athletes in the present study. Signals like the use of pain medication, braces, and tape, were considered important by athletes and sports physicians [4]. Many participants viewed these forms of (self) treatment as normal practice and they may allow athletes to perform adequately despite a beginning overuse wrist injury.

### Clinical implications

These disparities between athletes’ and sports physicians’ perceptions raise the question whether symptoms and functional limitations related to overuse wrist injuries are to be interpreted differently in young athletes. Clinicians and trainers are advised to be aware that young athletes may have difficulty differentiating between “a minor pain” and a beginning overuse injury. Both pain and limitations in daily activities and their impact on quality of life require the physician’s attention as indicators of injury. The items proposed here can aid sports medicine professionals in gathering appropriate information about a possible overuse wrist injury. VAS pain scores can vary widely among athletes and suffice only in part to identify overuse-related wrist pain. Descriptive aspects of pain like acute onset and longer duration, and any other accompanying symptoms could be addressed additionally. Furthermore, explicit questions could be asked about use of pain medication, tape, or braces, even when the athlete’s performance has not (yet) been affected by the injury.

For the assessment of limitations in daily life activities, many functional questionnaires such as the Disabilities of the Arm, Shoulder and Hand Outcome Measure (DASH) and the Michigan Hand Outcomes Questionnaire (MHOQ) have been developed [37, 38]. However, these instruments have not been designed to identify early injury but rather to evaluate injury outcome, and have mostly been developed based on input from adult populations. We therefore recommend physicians to ask specifically about the daily life activities that young athletes in this study considered important, when evaluating the possible presence of overuse wrist injury.

### Future directions

Various authors have suggested that overuse injuries in (mostly adult) athletes can be identified by means of questionnaires asking specifically about pain and functional limitations, instead of time loss [21, 39]. This study’s results emphasize that new studies on overuse injuries in young athletes, whether related to the wrist or to other locations, should focus on pain and limitations in daily and school activities besides sport-related limitations. For clinicians it is advisable to explicitly address functional limitations, and use of pain medication, tape, or braces during sports participation.

In order to help young athletes identify whether they should seek help because of a possible overuse wrist injury, we aim to develop a self-report questionnaire, the SOS-WRIST. The input of experts is required to compose a collection of relevant items and achieve adequate content validity for such a questionnaire [40, 41]. The items proposed in this study as well as those previously reported by experienced sport physicians can provide this expert-based input [4]. For professionals like trainers, coaches, and sports physicians involved with young athletes, such a tool can provide valuable information on young athletes’ experiences with (beginning) overuse wrist injuries. This may in turn contribute to earlier diagnosis and treatment of such injuries.

## Conclusions

Contrary to assumptions by sports physicians, young athletes consider pain and limitations during daily activities important indicators of overuse wrist injuries, while sport-related pain and limitations may not be reasons to seek help immediately. Factors that require physicians’ attention in young athletes with suspected overuse wrist injury include numerous daily life activities that cause pain or limitations, as well as acute pain onset, pain characteristics, and accompanying symptoms.

## Additional files

Additional file 1: Signals and limitations related to overuse wrist injury derived from focus group of young athletes (Additional file 1 – A hidden mismatch.pdf). (PDF 239 kb)

Additional File 2: COREQ (COnsolidated criteria for REporting Qualitative research) Checklist. (PDF 490 kb)

## Abbreviations

COREQ: Consolidated Criteria for Reporting Qualitative Research
VAS: Visual Analog Scale

## Authors’ information

Three of the authors (LK, GK and MM) work within the Amsterdam Collaboration for Health and Safety in Sports (ACHSS), which is one of the 10 research centres accredited by the International Olympic Committee (IOC), to improve prevention of injury and protection of athlete health.
