# Supplementary material for: A hidden mismatch between experiences of young athletes with overuse injuries of the wrist and sports physicians’ perceptions: a focus group study
Source: BMC Musculoskelet Disord. 2019 May 20;20:235. doi: 10.1186/s12891-019-2616-y (PMC6528344; doi:10.1186/s12891-019-2616-y)
Supplement: Supplementary file 1 — Signals and limitations related to overuse wrist injury derived from focus group of young athletes (Additional file 1 – A hidden mismatch.pdf). (PDF 239 kb) [file 12891_2019_2616_MOESM1_ESM.pdf]

Additional file 1

A hidden mismatch between experiences of young athletes with overuse injuries of the wrist and sports physicians' perceptions: a focus group study

Laura S. Kox, MD [1,4,5], Jip Opperman, BSc [1], P. Paul F. M. Kuijer, PhD [2], Gino M. M. J. Kerkhoffs, MD, PhD [3,4,5], Mario Maas, MD, PhD, [1,4,5], Monique H. W. Frings-Dresen, PhD [2]

Corresponding author
Laura S. Kox
Department of Radiology and Nuclear Medicine, Academic Medical Center
Meibergdreef 9
1105 AZ Amsterdam, The Netherlands
Phone: +31 20 566 29 05
Email: l.s.kox@amc.uva.nl

	Example verbatim quotes	Participant code
**SIGNALS**		
**Athlete characteristics**		
Physical characteristics		
1. Sex	*Yes, men reach their peak a bit later anyway and their peak period is longer. So for women this is usually around their 16th -18th and for men around their 18th-21th, but they will stay at that high level longer than women, so they have much more time to train and reach this peak.*	1
2. Age	*I have also put down age. Because, of course, when you are young, you still have your growth plate and such, naturally, and many athletes start having trouble with that.*	1
3. Hypermobile wrists	*That you can really see it, too. That is much better for you yourself, as well, that you can really look more easily, like with hypermobile and the like, you may not even know that, so that is tricky then, but you can already see and feel such things right at the moment itself.*	14
4. Carpal bossing	*And I have never really looked at it properly myself, so I do not know whether or not I already had carpal bossing then.*	14

Injury history		
5. Previous wrist injury	*Yes, you start thinking about it, of course, and a year and a half earlier I also had wrist complaints once on my right wrist, but that was with a completely different sport and not at all with the same movement, and yes, that had just disappeared. But anyway, you do start thinking back that that may have had something to do with it. Still after such a long period.*	11
6. No history of wrist injury	*And yes, I have never had trouble with my right wrist, so I did not know what it was, whether it was serious.*	2
7. No history of serious injury	*And then I thought, well, I have never really had a serious injury, so, well, it will surely not be all that bad.*	2
Sport-related characteristics		
7. Change/adaptation in technique (because of other injury)	*Then I started having trouble with my left wrist. Then I thought, well, yes, I am going to play single-handed. Then you can exclude it and you do not need it. But then my right wrist started bothering me.*	12
	Your right arm you could effectively exclude, actually. So that you started doing everything with the left, but that one also started to be overloaded then, of course.	11
8. Increase in training intensity	*It was after a three- day training camp, so I had been rowing quite a lot then. And there was a lot of wind during the training camp, and afterwards it turned out that that could have been a cause for my complaints, I think.*	3
9. Increase in training frequency	*I had a tournament last summer with [xxxx] then, and then you play rather a lot of matches and then in one week you have only once without a match, so to speak, and then you train pretty much double.*	4
10. Change in training content	*Just after the summer I then switched to the full-time JTS group. Then we had trained more and worked a lot on grip for two or three months.*	10
11. Change in training group	*And around the new year, we had a training that did not go so well and there was a lot of wind and a new guy in the boat and, I don't know, a lot of commotion.*	5
**Symptoms**		
Pain		
Quality of pain		
12. Pain	*For me it is not really a nagging feeling, or just sensitivity, but for me it is just real pain and discomfort.*	15
	Yes, I think that the connotation of pain is also just that it is worse than discomfort. Like yes, I also feel discomfort in my leg, because I have slept with my leg in a twist, or something...	11
	And I noticed myself that I also wanted to look for the pain, or something, very weird, but that I wanted to find out, does it still hurt?	3

13. Pain different from normal		
14. Annoying pain	*Yes at his moment, it is just such a constant annoying feeling, I would say.*	11
15. Irritating pain	*But it was not that I was screaming with pain. It was just very annoying, and every now and again I would think, just stop it, it is so irritating!*	3
16. Pain as a bruise	*Yes, at first it was just, yes, every time when the ball landed on it, yes, just like a bruise when you press it, that kind of feeling.*	17
17. Nagging pain	*But I have also had for a long time that it is nagging continuously, then it is around 2-3 [on a scale from 1 to 10].*	6
18. Radiating pain	*Every now and again, that it really goes all the way through.*	14
19. Shooting pain	*And yes, I still have trouble with that, that I get these shooting pains.*	14
20. Sharp pain	*Yes, no it is a sharp pain.*	4
21. Stabbing pain	*Yes, it really is a stabbing pain and it continues to hurt, it does not go away.*	12
	But, for example, stabbing pain, if in rest for me, for example, it is also a stabbing pain, then I would really think, oh that is not quite right.	4
22. Feeling dull from pain	*It differed. When it was really bothering me, it was really an 8. Then my whole arm was just hurting and that I was really feeling dull because of it.*	15
23. As if whole wrist is pulled apart	*It seems as if your whole wrist is pulled apart.*	18
Timing of pain		
Onset of pain		
24. Gradual onset of pain	*It started, yes, it started, let's say, very slowly, I think a year ago, a year and a half ago perhaps. I do not know all that well when it started.*	8
25. Sudden onset of pain	*Well, I was rowing, and I suddenly felt a pain in my wrist.*	3
26. Acute cause of pain	*Yes, well, according to me I was doing something on the floor, I was making a movement and then, yes, I was bothered by it and that continued during that training, but I thought, oh well, it is probably not that much.*	16
27. No acute cause of pain	*No, that is what I thought was the very worst. Because I have always, when I have pain, I like to know where it comes from. And that I did not have. I still don't.*	20
28. Onset after training	*During a technical training with a lot of resistance it felt then as if I hit my 'funny bone' but not in my elbow, but in my wrist. Yes, that, shake it off and go on.*	11
29. Onset during competitive event	*Yes, during the match my wrist suddenly started bothering me.*	17

30. Onset after competitive event	*Yes, I had a match, and suddenly afterwards I just had.... not during the match, I had discomfort, only after the match it suddenly started to bother me. And then, yes, I only needed to judo once more, so I still did that. And then, yes, then it actually stayed, all the time.*	19
31. Onset in period of rest	*And then afterwards it came up, actually, once I started to rest, then I had a week off, that is when it really seemed to loosen up, then I felt it a bit more.*	4
Course of pain		
32. Intermittent pain	*Well, it was not really a pain that I had throughout the day.*	3
	Yes, usually it tapers off after a while and then it comes back after a while. So actually quite permanent. So it is not as if I am completely free of it.	10
33. Continuous pain	*In fact everything was just hurting.*	5
	Bearable. But continuously, and that is very tiring, continuous pain.	6
34. Persistent pain	*It stayed, certainly with volleyball land the like, if I played or something I actually had pain immediately.*	14
35. Progressive pain	*Yes, it seemed to become less at first, but afterwards it came up again, probably because I had made a wrong move or something, or that I started doing too much again. And then it became much worse again.*	15
36. Increasing pain with loading	*Then it became worse all the time, yes, the more you stood on your hands, the more you were resting on them, the more it started bothering you.*	1
	And then the next day, actually during an intensive training, then the pain started to become much worse.	11
37. Pain with repetitive movements	*It bothers me especially when I do the same activities one after the other.*	14
Duration of pain		
38. One day	*No, yes at the end of the training, then you would go to sleep and then the next day you would wake up and it would be gone again.*	1
39. Three days or longer		
40. Longer than a week	*After a week it was still not gone.*	3
41. Two weeks		
42. One month	*A month maybe. If you have it all month, every time, but I would sometimes have it for a week and then it would be gone for a while. So yes, then I do not go. Then I wait.*	8
43. Longer than one month	*Yes, of course, I have continued for six months, so I am now saying two weeks, but I have continued for much longer, of course.*	6

Moments of pain		
44. Pain at night	*Or during sleep itself.*	1
	So it depends a little on how much pain I have during the day and at night as well.	20
45. Pain in the morning	*And then even worse, if I had been lying on it, that you would really wake up with a kind of stabbing pain*	8
	Because really, that I would be waking up and I would be thinking, oh no, this is not right.	15
46. Pain with cold	*That when it was cold during December and November, if we were playing hockey then, that I really just, yes, as you were saying, needed to tape it up, so that you cannot go any further than a certain point. Because it just hurts much otherwise.*	7
47. Pain with heat	*And that mainly had to do, I thought myself, with cold and heat.*	7
48. Training event soon		
49. Tournament soon		
50. Championship soon		
51. No pain during rest	*Yes. So when I was just sitting like this, I did not have any pain, only if I started doing something, then I did.*	2
	Well, during the vacation, then not really.	18
Pain during rest		
52. Pain during rest	*No. Well, yes, I do if I have trained really hard the day before and that it hurts during rest as well.*	16
53. Annoying during rest	*Yes. The pain was really not an 8 then, but just very annoying.*	3
54. Nagging during rest	*No, I often did not have it during rest, but I can, suddenly I remember again, that I was visiting with a friend and that we were just normally watching TV, and that I then I think just for an hour, or so [...] that after that really after that I kept having a lame feeling, that was, not those stabbing pains, but really just a feeling that I kept thinking, oh dear, that I was sitting like this, let's say [is holding wrist].*	3
55. Pain score 4	*But yes, now, it just depends. Sometimes it can start stabbing, but yes, then it is, if I do nothing, I think it is a 4 or something.*	14
56. Pain score 5		
57. Pain score 6	*When I did nothing, I had.... If I had to give a score, when, at the time, I would be doing nothing, then I really had a pain threshold of 6 to 7. When just nothing at all, for example sitting in the car and doing nothing.*	13

Severity of pain		
Pain in words		
58. Never have felt a pain like this	*Yes, it just started, I was hitting a backhand, suddenly you feel a pain I had never felt before. Really, like, well maybe it will pass.*	12
59. A lot of pain	*And then I was really aware that I had to stop that, because it really hurt a lot.*	5
	Well, I just first had really a lot of pain, that just after a training or after lifting something heave, that you think, there is really something wrong there.	20
60. Crying because of pain		
Pain in number		
61. Pain score 1 to 2	*Yes, just irritating.*	7
62. Pain score 3	*But I have also had for a long time that it nags continuously, then it is something like 2-3.*	6
63. Pain score 4	*Yes, 4 or 5. Not really that I could not, like, wit hit. I could do everything normally, only yes, it did hurt. But not that I could not do things anymore because of it.*	8
64. Pain score 5		
65. Pain score 6	*Well it was not at its worst, really, I think a 6 or something, and then, well yes, I put some tape around it and then it goes down to 4, 5, that it just, that you can continue, let's say, that you do not need to stop.*	1
66. Pain score 7	*Now it is not so bad, but, of course, when I have played I do really notice it. Then I think it is sometimes still 7.*	14
	During rest it does not bother me and while training, a 7 I think, sometimes. When I really am in pain.	21
67. Pain score 8	*The times that I really could not bend it, and then it would just bend accidentally, then it would be a 7 or an 8.*	6
	The day after? I think also an 8.	9
68. Pain score 9	*Well, if it really is very painful, an 8 or a 9, perhaps. Because then I really have, that I think, now I should really not do anything for a while.*	20
69. Pain score 10	*Yes, I think really around 9 or 10. Yes, I could not do anything anymore.*	9
	The times that it was very bad, then I really think it was a 10.	11
70. High pain score		
Location		
71. Dominant side		
72. Non-dominant side		

73. Pain in whole arm	*And I very often had just in the evening that I had done a lot of done heavy lifting or… that my whole arm instead of my wrist was really bothering me. That it just radiated through my whole arm.*	15
74. Pain in hand and wrist	*If he says, where is the pain, and yes, it actually is in my hand, but also in my wrist. So yes, he does not know whether it is your hand or your wrist. Or maybe a bit further in your lower arm. [...] Yes, exactly, it is not a specific area.*	12
75. Pain in radial side of wrist	*A stab. Especially if I did this, let's say. This movement and then I just felt a stab. [Points out: first dorsal compartment on ulnar deviation].*	15
76. Pain in ulnar side of wrist	*So there I feel it very much here on the side [points out ulnar].*	4
77. Pain in middle of wrist joint	*Then I feel it a bit more in the middle, too.*	4
78. Pain in dorsal side of wrist	*Yes, for me it really was mainly on the upper side, actually, or well, yes, more inside the joint really. [Points out: dorsal side wrist] But you felt that more on the upper side, yes.*	1
79. Pain in ventral side of wrist	*Yes, it is here, let's say, a little, and then, let's say, really in the wrist. [Points out: ulnar and ventral side wrist]*	4
Rowing		
80. Outside hand (closest to end of oar)	*It was on my outside hand. I was sitting starboard. And then it was really strange, actually, that my outside arm was bothering me, in principle you keep that one straight and with the inside hand you turn. So it is very strange that I actually started having problems with my outside hand.*	11
81. Inside hand (closest to rowlock)	*Yes, then it hurt as well. Especially when turning my oar, because I row portside, so I have my oar on that side, so my right hand turns my oar, well yes, and that was where the injury was, too.*	3
Pain with specific activities		
Pain with specific movements		
82. Pain with all movements of wrist	*But that way it bothers me the least, because with every movement I make I actually have pain, or feel, or can feel.*	20
83. Pain with ulnar deviation of wrist		
84. Pain with dorsiflexion of wrist	*Yes, I, especially like this and like this, because then you really apply a lot of pressure and force. [dorsal and ventral flexion wrist]*	20
85. Pain with hyperdorsiflexion of wrist	*Yes, just when it is overstretched, towards the back or the front, then it hurts.*	10

86. Pain with palmarflexion of wrist	*At this point if I just bend normally, then in between here, at the middle finger bones, then they hurt... Metacarpal bones. And then, yes, it is just as if something is just pushing against the bone, or something.*	10
87. Pain with hyperpalmarflexion of wrist	*Only with those yes, extreme bends, or how do you call them. Only then, really.*	4
88. Pain while turning the wrist	*A bit of a stab, let's say here, and while turning especially. [Points out: ulnar side wrist] And yes, during games especially, yes.*	19
89. Pain while grasping		
90. Pain while squeezing	*Just stabbing pain, just like this back and forth, and when I often squeeze it and such, then it just hurts.*	12
91. Pain while catching something	*And that could be triggered by doing push-ups, or for example by catching something. A door that you just catch, or things like that.*	6
92. Pain while catching oneself after a fall	*Yes, that depends a bit, I actually only have complaints if I fall and catch myself.*	7
93. Pain while stabilizing wrist	*It was mainly, with me, while stabilizing my wrist, that instability, that is rather extreme, I understand, in my wrist.*	6
94. Pain while hanging		
95. Pain while pushing		
96. Pain while pulling		
97. Pain while flexing thumb		
Pain with activities of daily living		
98. Pain while washing dishes		
99. Pain while cutting	*For example while cutting, if you make the same movement all the time, then it also comes back again.*	14
100. Pain while lifting heavy objects	*And for the rest I still had trouble with lifting heavy things.*	8
	No, this way it is all right, with the splint, then I hardly feel anything. But even if I sit like this and I would have to lift something heavy, then I feel it stabbing.	20
101. Pain while picking up (heavy) objects	*So you can pick up a pitcher like this, and then your whole hand bends down. [...] This I feel already, I feel now that it is being pulled apart there.*	6
102. Pain while pushing up from chair or bed	*But now, for example, really a second ago I was pushing myself up to sit up properly and then I feel a little stab. But if I do it for longer, I do really feel a bad stab.*	4
	Yes, at a certain point, when it was also really bothering me during volleyball, then I also had a problem with just lifting things, leaning on my hands if I wanted to get up.	17

103. Pain while painting	*A while ago I had to paint part of my room and then this thing has to be finished, and when I keep making the same movement all the time, then it also begins to become quite sensitive.*	18
104. Pain while using phone	*Yes, no, sending just more than one Whatsapp, then I felt it again, too.*	17
105. Pain while grasping handle		
106. Pain while grasping doorknob		
107. Pain while driving		
108. Pain while riding bicycle	*Well yes, that did not go well, because even while riding a bike it was bothering me. If I just rode across a bump of, let's say, 2 centimetres, or something, then I felt the vibration through my wrist.*	13
109. Pain while riding bicycle standing up	*Well, so it was just purely when I lean on it, so also while riding a bike standing up. Normally, with those kinds of things.*	16
110. Pain while walking	*Yes, during my daily routine I wear this thing especially, because I have the least discomfort then, but if I would not wear it, I would be in pain with everything I do, writing, typing, walking, then also while picking up things, lifting, actually with everything.*	20
111. Pain while brushing teeth	*And when I was brushing my teeth, at some point that really started to hurt super-much.*	3
112. Pain while combing hair	*For example, when I was brushing my hair, then I would suddenly have a stabbing pain there.*	3
113. Pain while washing	*Dressing, towelling dry, those kinds of things*	5
114. Pain while getting dressed		
115. Pain while blocking door		
116. Pain while accelerating on motorscooter		
Pain with school/study		
117. Pain while carrying school bag	*And when lifting things, like my bag, like this.*	18
118. Pain while typing	*Typing, actually everything just hurt.*	5
119. Pain while writing	*And while writing, then it really bothers me, right handed. If I need to write a lot, then I my right wrist also starts hurting.*	12
	Then before, a lot while writing, that did not work, so I learned to write left handed.	14

120. Pain during gym class: pommel horse vaulting	*Yes, and while doing gym I also...Yes, but then we do pommel horse vaulting, or something, and then I also feel it a bit.*	8
Pain during work		
121. Pain while carrying plates (waiting tables)	*I work as a waitperson. So yes, often while stacking plates and those kinds of things I also do feel it.*	10
Relation with sport: general		
122. Always pain during sport	*I never play without pain.*	12
123. Pain during bench press	*When I do fitness, I also have trouble benchpressing. And then the day after I really cannot do anything with my left wrist.*	12
124. Pain while hanging and pulling up	*Also with push-ups or hanging from the bar.*	18
125. Pain during push-ups and core stability training	*While doing push-ups, for example it really bothers me already, then yes, my wrist sort of doubles over completely, and if I put pressure on it then I really feel it very badly.*	4
	I really have often had problems with my wrist. Already with push-ups, then, those kinds of things.	6
126. Pain during training	*Because my wrist only became more and more swollen and painful.*	11
127. Pain during (practice) game	*Because during trainings I just almost have no trouble, only at the end we play some games and then it starts bothering me again.*	19
128. Pain after training	*But now it is actually limited to the evening itself that it hurts, and sometimes the next day.*	14
129. Pain after game	*And then after the match it comes up, you know.*	4
Relation with sport: volleyball		
130. Pain with every ball on wrist	*Yes, because you get many hits on your arms, because actually everything you do is with your arms, so yes. [...] And then with every hit, then you do feel a kind of stab shooting through your arms.*	20
131. Pain with service	*And yes, I am right-handed, too, so I had extra discomfort. Because also when you serve and smash, you use your right hand.*	20
132. Pain with smash		
133. Pain with underhanded movements	*Well, in the beginning only while playing underhanded.*	17

134. Pain with overhanded movements	*Especially playing overhanded. It actually doubles up also quite often, backwards. That it stretches back a little too far.*	14
	But I do feel with overhanded movements that if you really need to play that way, that it really begins to bother you a lot more, because that way there is much more pressure on your arm.	20
Relation with sport: field hockey		
135. Pain with everything in field hockey	*No, I noticed it actually all the time.*	15
136. Pain while hitting the ball	*Hitting, just yes, making a hitting movement with the stick, because you are holding it in your hands all the time.*	15
137. Pain while slapping		
138. Pain while pushing	*But for example, during indoor hockey, then you do not hit, or slap, then you only push. And then I do have like, oh well, this I should not be doing for a while this weekend.*	7
139. Pain while moving ball back and forth		
140. Pain while stopping ball		
141. Pain during disguised slap	*Well yes, for example the disguised slap, that is when you really just, sort of double up completely and you hit, let's say, straight through and play it really in that direction, so then you have to really bend backwards quite far and that does really hurt a bit, actually.*	4
Relation with sport: judo		
142. Pain during turning movement	*Yes, just everything more or less. Just you....when you put a little pressure on and during turning movements and so on.*	19
143. Pain while pulling	*Well, when putting on pressure, or yes, especially pushing or pulling. You do that constantly in judo but.....*	18
144. Pain while strangling	*Well sometimes it does really hurt, when you are sitting on the floor and you want to strangle someone. But then you do not have the power to pull and then you have to let go, and that really does hurt.*	18
145. Pain while holding (lapels)	*As soon as it hurts, then you just let go of something of course, so that you do not.... yes, you have no grip any more.*	10
146. Pain while lifting someone	*Unless you need to lift the person in front of your, that's when you feel it again.*	18
147. Pain while breaking fall		

148. Pain while pushing	*Everything that, everything I really needed to also do with force, also that pushing position, but also really with force, if that involved moving before the throw, that could also be really difficult.*	6
149. Pain while supporting body		
150. Pain while walking on hands	*Yes, just the hand-stand and then it is at a 90 degree angle, that my wrist cannot do without pain, I assume. I think. Yes, if a lot of pressure is put on it, that also still bothers me.*	13
Relation with sport: gymnastics		
151. Pain with exercises and landing on (hard) floor	*Yes, during gymnastics you have, let's say, that floor is not very... like a trampoline, let's say. It becomes harder. So if you land on that, for example, from a high jump, on your hands, that impact that it takes then, let's say.*	21
152. Pain with parallel bars exercises (hanging)	*And with other exercises on the parallel bars, because you then every time, yes, you hit those bars quite fast with your hands.*	21
153. Pain while doing back handspring on beam	*And then I was having more and more pain and so on, while doing back handsprings on the beam, because that is quite hard, a beam like that, let's say.*	21
154. Pain with pommel horse exercises	*[other participant:] and you also have, that is called the pommel horse, or something? And then you also need to, let's say, push off with your wrists?*	
	Yes. Then I also had problems all the time.□	1
155. Pain while leaning	*Yes, when you leaned on it, it hurt.*□	1
156. Pain with handstand		
157. Pain while jumping		
Relation with sport: tennis		
158. Pain with backhand		
159. Pain with forehand	*Yes, that forehand was really just, really very much pain.* □	2
160. Pain with spin	*If you use spin, then the ball, it slows down. You can hit very shallow, but then you cannot it hard, because it will be out. But if you hit had and use spin, then the ball will still be in while you are hitting hard. Only then you have to turn all the time with your wrist.*	8
161. Pain with volley	*Yes, with volleys, because then your hand often bends backwards, even though you actually do not want that, but it just happens then.*	8
162. Pain while hitting ball the wrong way	*Since then it actually only bothers me if I hit a ball completely the wrong way. So then, normally the ball bounces with your [racket] tension, but if you hit it the wrong way, then you feel such an impact in your wrist and then you do feel it for a bit and then it goes away again.*	8

163. Pain while holding racket	*But I just went to find out, swing my racket without touching a ball or anything. But then I still felt pain, actually, so then I thought, well, still not all that good.*	2
Relation with sport: rowing		
164. Pain while on rowing machine	*The rowing machine hurt less. Yes. That was not too bad at a certain point. Even though it still was not gone, let's say. And I also think that is because there you need to bend your wrist less.*	3
165. Pain with hanging on outer arm		
166. Pain when turning oar	*Yes, turning the oar, that is wat it is all about. It is my inside hand [closest to rowlock].*	5
Treatment		
167. None / continue sport	*Then it just bent double like that. And then, yes, then at first I just continued all the time, while I did have just a real bump on my hand.*	14
	No, because I think that I have also continued a bit too long, with volleyball itself already. Because after the diagnosis of tendonitis I continued to play volleyball for at least a few months and I think that my complaints have been worsened because of it, because I thought, yes, there is nothing that can be done, I thought, so I might as well go on. Perhaps I should have stopped at that point, or taken it easy, in any case.	20
168. Rest	*So then I think I did not row for a week or so, or perhaps even two. [....] And then at first I gave it a rest for a week, and then started again, and then pain again, and then off to the doctor.*	5
	I started by giving it a rest, tried again, still painful.	12
169. Ice	*And cooling, every now and again, just to reduce it a bit.*	14
170. Medication	*Yes, when you think that it is an inflammation, let's say, that you then just pop a few diclofenac, or something, but...*	1
	Well, as such, if I can make a movement, but if it hurts, then I can also, without a problem, then I take a painkiller and then I can still do it, so then I just do it.	4
	So no, then I always used to tape it up and I would take one of those pink pills and then it was bearable again.	7
171. Tape	*So yes, what I actually used to do was tape around it and then continue with a little less pain.*	1
	But then, if it is too bad, then I just put a little tape around it, so that I do not have to think about it, let's say.	4
	Then a bit, yes, starting by taping it up myself, and so on. It did work.	12

172. Brace	*Yes, I also have to say, I have also used a brace at a certain point, that was to alleviate the load on my wrist, that I could move it less freely.*	2
	So then I bought a brace.	15
173. Strength training	*After I had gone through my wrist, I also started power training. That really did help a lot.*	9
174. Physiotherapy: exercises	*So then I went to the physio for it, and he said that it was overuse, so then I had to do exercises.*	16
175. Physiotherapy: massage	*There is a physio downstairs where you can go ahead of time or afterwards, they turn something, they turn something and massage it a bit, and yes, a bit.*	19
176. Corticosteroid injection	*I have had one of those steroid shots and now it has discoloured a little.*	2
Effect of treatment		
177. Pain remains despite medication	*And afterwards, taken the diclofenac, but it did not get any better.*	3
178. Pain remains despite rest	*But then I did not play volleyball for a while, but the pain continued there all the time.*	14
179. Pain decreases with rest	*But that really went away within a few days as soon as I was not playing hockey.*	4
180. Dependency on brace		
Symptoms other than pain		
181. Snapping	*I just hit a forehand, actually, and I felt a kind of 'snap', actually.[...] yes, I felt really just a kind of whip-like something, you know.*	2
182. Clicking		
183. Cracking	*And also, I was at the doctor's recently and then I just sat like this, let's say, and then if I turn my wrist it also cracks [cracks with wrist]. And so I have that every time if I turn it around, and that was not right, or something, they said.*	21
184. Creaking	*Because it creaked, let's say, inside my wrist also. But really, if I did this, it was really just real creaking, and I could also sort of hear it, let's say.*	3
	And it creaked a lot, too. So you could make it creak easily if you wanted to and moved it.	18
185. Grinding	*I would really call it a grinding noise. Mine always makes a grinding noise.*	4
	Then it was.... then it also made a lot of grinding noise and everything hurt.	5
186. Rough feeling inside	*Yes, if I did this, then it felt very rough inside. Yes, a rough feeling. I do not know if you really hear it, I have never really listened...*	3

187.	Discomfort	*Yes, I think that the connotation with pain is also just that it is worse than discomfort. So like, yes, I also sometimes have discomfort in my leg because I slept the wrong way, or something...*	11
188.	Pressure	*You are just aware of it. So yes...A kind of pressure.*	11
189.	Presence	*But now, yes, not real pain. But it is present all right.*	5
190.	Nagging	*Yes, how would you describe that? Yes, that it nags a bit, or something. As if you have been kneeling for a long while and that you then, yes, I don't know, how do you describe that? No idea. There is something there.*	1
		Yes, nagging, too. Just that you say, well, what is that, you know.	3
191.	Stabbing feeling	*And when you are doing something really heavy, then you really feel it stabbing.*	20
192.	Irritating feeling	*An irritating feeling, or something.*	1
193.	Feeling of muscle acidification		
194.	Stiffness	*Sometimes I think, it is stiff and then 'snap', it is all right again. I also have sometimes, often just after I wake up, then they are really stiff and then 'snap', then I bend them...*	12
195.	Jamming	*No, it feels different now, it feels, let's say, as if it continues to, as if it is jammed and needs to be loosened up, let's say.[...] And now I really have the feeling it is jammed and cannot move optimally, let's say.*	21
196.	Instable feeling		
197.	Swelling	*But it did not go away, and my wrist also became quite swollen.*	3
		But so it had, in the beginning, just become really thick and swollen.	2
		It just swelled up all the time.	14
198.	Bump	*Yes, it is here, a bump, right here, on my wrist, and that is not supposed to be there. [points to dorsal side of wrist]*	20
199.	Redness	*But, actually after the first time I had it, then I did see that it was there, it was swollen and red, that is not a good sign, of course.*	5
200.	Discoloration	*And yes, that was a little blueish, red, and swollen.*	15
		Yes, it was just totally blue and swollen. Yes, it just felt...yes, it began to bother me, but I thought, oh well, yes, a bruise.	17
201.	Warm		
202.	Reduced strength	*Yes, It did not feel strong.*	18
203.	Shocks through arm		
Limited movement			
204.	Some limitation of movement		

205.	Complete limitation of movement	*Yes, I just could almost not move my wrist.*	2
206.	Limited dorsiflexion	*Yes, because now I cannot go further than this, and I used to always be able to. [dorsiflexion]*	21
	Timing of symptoms		
207.	Started suddenly	*I have [...] gone through my wrist in the semi-final. Then it said 'snap'.*	9
208.	Increased with loading	*Well, after the match, for example, because then, of course, yes, you must give a top performance. Last weekend. So then it swelled up again.*	5
209.	Progressive		
210.	Intermittent	*Well, actually I forget it usually halfway through the rowing. Then I thing, oh yes, oh yes, my wrist. But that is a good thing, too.*	5
	Relation with sport: gymnastics		
211.	Leaning	*So not that it is stretched here, or something, but really, yes, leaning itself*	1
212.	Handstand	*We also have to do handstands, but then, because you are standing like that for so long, you get such an overstretched feeling here [points to dorsal side of wrist].*	3
	Relation with sport: tennis		
213.	Forehand	*Now I play almost always without pain. Only sometimes with my forehand if I myself need to accelerate, let's say, that now and then still a little bit, yes, I don't know, it is not pain, but a little unstable, sort of. So that, a little bit.*	2
	Location		
214.	Whole arm	*And my arm became just really completely swollen. It was really, my tape was just much too tight. I think that my arm was about twice as thick as it is now. At the time.*	11
215.	Whole wrist	*Yes, I always feel it, but it used to be, then I had a very swollen wrist for a week and then, if I started to play volleyball again, then it was gone again and then I started to play volleyball again.*	14
216.	Dorsal side of wrist		
217.	Radial side of wrist	*No, it was here, really on the side. [Points to radial side] and it did creak.*	3
218.	Ulnar side of wrist		

Treatment		
219. None / continue sport	*Then it just buckled, like this. And then, yes, then actually at first I just continued all the time, while I really did have a bump on my hand.*	14
220. Rest		
221. Ice		
222. Medication		
223. Physiotherapy: exercises	*So now I do exercises, too, squeezing and so on.*	18
Effect of treatment		
224. Symptoms decrease with rest		
**LIMITATIONS**		
Limitations in sport		
Competition		
225. Unable to use wrist like you want during competition		
226. Miss competitive event	*Your opponent can be very strong, or you get a lot of hard balls and then you have to hit very hard, too, so then I actually, it was during the summer vacation, so then I actually called of my match.*	8
	But you always start doing things during a match that are different from what you do in a training, too. You always try harder to find your limits.	9
	Well yes, the past few weeks, a year and a half or something, almost none. Yes, team matches and so on, I tried to do in the beginning, but yes. It just does not work. You just cannot judo full tilt, let's say, so that does not go well at all.	19
227. Give up during competitive event	*And then I júst lost the set, and then it was really just, OK, I give up, I am going to stop. And yes, I actually never stop, I have only given up once, I think, a long time ago, because I had a cramp, I think. So it was one of the first times, sort of.*	2
	So I could not use my hand any more. So yes, then I just called off everything after all, I just stood on the mat to salute. So then I just gave up.	9
Training		
228. Stopped sport	*I do not do it anymore. I have, since that pain, since I have really serious trouble, I have, I think, at least six months, I have stopped. And now I really want to pick it up again, but I have not had a chance yet.*	20

229. Unable to use wrist like you want during training		
230. Stop during training / adaptation of training load	*So then I stopped training and the next day I went to a physio.*	11
	But it, for example, with training, if I had problems, I said, well guys, I am stopping for a while, because my wrist is beginning to bother me.	15
	Yes, usually I do a little less. Then I do it three times, instead of twice.	16
231. Skip training sessions/ adaptation of training frequency	*And I also started just to train less.*	12
Adaptation of training content: skip parts of training		
232. Push-ups	*With push-ups, for example, then I just go and do a plank, or I do some equipment where you do not lean on your wrists.*	4
233. Biceps curl		
Adaptation of training content: alternative training components		
234. Strength training	*I still do not put force on my wrists now, I only do on my legs.*	17
235. Sit-ups	*No, but I can do sit-ups, so that works all right. Then I just see what I can do.*	18
236. Swimming	*I have also done swimming for a long time and that worked all right.*	14
237. Running	*Yes, just running, and what else did I do, well, mainly running.*	3
238. Cycling	*Yes, but still, for me it was also, well you must keep fit. I have just used the spinning bike 4, 5 times a week for an hour and a half. And then without leaning forward, because then you put a load on your wrist again, just sitting up straight, just to maintain your condition.*	11
Adaptation of parts of training		
239. Bench under bar when hanging	*Normally you turn a bit also with your elbows, only then it tenses up completely, so to speak. And yes, but if you then put a bench underneath, then you do not need to stretch all the way.*	19
240. Push-ups on fists	*I prefer not to push up, like this, then I do it either on my fists, or I put my hand down differently, or I don't know.*	7

241. Core stability on elbows	*If I, for example, then I need to lean on my wrists for a long time and then sometimes I just stop the exercise, then I do it in a different way, on my elbow, for example. Because I cannot stand on it any longer.*	4
	No, yes, doing the plank on my elbows, but not on my hands.	17
Gymnastics		
242. Fewer back handsprings on beam	*And yes, as little as possible doing back handsprings on the beam and those kinds of things.*	21
243. More on soft floor	*Well, yes, a lot, let's say, on the floor, very many series on soft, that is the nicest, let's say, not just on a hard floor.*	21
Adaptation in technique		
244. Compensation movement		
245. Technique is not possible anymore		
Adaptation in technique: gymnastics		
246. Changed sequence of elements	*I always do a cartwheel and a summersault one after the other on the bench and because I ...[laughter] ...because I lean on my wrists for a cartwheel, I started it a little more carefully, so we then split it, so that I did not need to do them one after the other.*	16
Adaptation in technique: volleyball		
247. Not playing overhanded	*And yes, overhanded I am not allowed yet, because they think that there is the biggest cause.*	17
Adaptation in technique: tennis		
248. Play fewer forehands	*Only because I started avoiding it a bit, just trying to do short rallies, so really just a bit of 'all or nothing' playing, a little bit of getting around doing the forehand, something that was really strange, because my forehand is better.*	2
249. Play fewer backhands	*Yes, I do try to hit backhands as little as possible. But yes, in matches you cannot avoid that. And then, yes, you just try to do it as little as possible, but sometimes you cannot get away from it.*	12
250. Play singlehandedly	*Then I thought, well yes, I will start playing singlehandedly. Then you can give it a rest, you do not need to use it.*	12
251. Hit less hard	*So then I also had to hit less hard again.*	8

252. Do not give spin anymore		
Adaptation in technique: judo		
253. Judo with other hand	*I judo on the left, but I can also start to judo on the right, for example, a little... Because at first I only had problems with my left wrist, so then you could do something, if you started to judo on the right, then that alleviated, let's say, a little, but… Yes. Still it is hard to….*	19
254. Change grip	*So yes, tried then to change my grip that I started grabbing on the back, but yes, I am really not a backgrabber. I am actually small.*	9
255. Push up on fists	*So I actually unlearned that really fast, by just doing it always on my fists.*	6
Adaptation in technique: rowing		
256. General technical adaptations	*I do have the idea that I have now found a way to row so that it does not really get worse, but it has not gone away.*	5
257. Elbows more outward	*And I try to really make sure that I turn my elbows outward properly, instead of just a little bit like this. Which is actually also good for rowing.*	5
Performance		
258. Put team at disadvantage		
259. Afraid to go all out		
260. No decrease in performance		
261. Decreased of performance or level	*Well, you put less of a load on it, but you also do notice that your level is going down.*	12
Limitations in movement		
General		
262. Unable to move wrist optimally		
263. Unable to turn wrist	*But yes, I could not do a lot of things. That turning, too, that did not work anymore.*	9
264. Unable to move wrist	*Yes, yes, then for a while everything. Because, yes, your whole hand actually just did not work anymore.*	5
	And then at a certain point, I think about a year ago now, then I woke up, and I do not know what had happened, but I had been sleeping in a strange posture, or something, then I rode the motor scooter to hockey and I thought, well, what is happening to me now, I cannot move my wrist.	7

265. Unable to extend wrist		
266. Unable to bend wrist	*So I could actually not really bend it. That I could really could not, something...That I just had to keep stable, or else it hurt. Yes, both directions.*	6
267. Unable to squeeze	*It was both turning and squeezing movements, not just one of the two.*	11
Sport-related		
Volleyball		
268. Aim ball	*But yes, I cannot move it backwards as much any more, so I just notice in playing that I play with left much more, so that the ball sometimes a...That I use more force with left, so that the ball goes in the other direction.*	14
Tennis		
269. Forehand	*So I could serve, I could hit a backhand, but I really could not hit any more forehands.*	2
Gymnastics		
270. Hanging on parallel bars	*And hanging from the parallel bars I could not do any more and I just could also, yes, I could not lean any more or whatever.*	21
271. Supporting body		
272. Handstand	*Well yes, when you had to stand still on your hands, so just some random exercise, then you did notice that you could no longer stand on your hands for any length of time, that you would fall back, because you could not flex your wrist completely, as it were.*	1
	Then at a certain point then I really could not do anything anymore. Yes, I could do everything with my legs, but if someone asked me, do a handstand, usually I stood for a minute, but then I came down after 10 seconds because I could not do it anymore.	21
Judo		
273. Walking on hands	*But now and then I still notice, like, oh, that is not going too well. For example, now and then exercises, then we have to walk on our hands, well, that I can still not do well.*	13
274. Pushing	*I still cannot really stop someone with my wrist, because then I have the feeling that it will buckle again and then I get those tinglings again and it feels like my fingers are numbing, that is how it feels.*	9
275. Loosening someone	*Well, I can still grab someone well, but yes, you can also loosen up, and so on. Yes, some people they put, that is just their style of judo, but there just also are people who do that on purpose, but they, yes, they just loosen that hand all the time.*	19

276. Limitations of dominant hand	*But it makes a difference that I am right handed, so I move much more with the right and yes, left, the only thing I need to do is holding the sleeve and then just, hold tight, so I actually do not need to move my wrist any more than like this, actually. So that went pretty well.*	13
277. Many throws	*For judo that is... is all about the wrist. If you cannot move your wrist properly, yes, then there are very many throws that you cannot do.*	13
278. Pulling	*I could just not keep up the power in my hand on the side of the lapel.*	9
279. Tying band of judo suit	*So yes, then I still stood at the warming-up room again and then I was tying my band and I did not manage anymore.*	9
280. Breaking fall	*To break your fall, to transfer the energy onto the mat. And the harder you … the better you fall. If you are just not really warm yet, and you think just really …, and… Yes, that was at a certain point that I could not really fall on that side, because I could not properly … anymore.*	6
Activities of daily living (ADL)		
Limitations in ADL		
281. Unable to use wrist	*I also had a lot of trouble with it in my daily life. At a certain point I could not really function with my whole right arm, actually.*	11
282. Unable to use wrist the way you want		
283. Combing hair	*Well yes, I could not brush my hair either.*	2
284. Brushing teeth	*And then I could not brush my teeth any more.*	5
285. Going to toilet	*And also going to the bathroom, or something, all sorts of stupid things, yes, I could just hardly move my wrist.*	2
286. Getting dressed	*Then I had to wear a different suit, because I was wearing a blue suit and I had to be on the podium in a white suit. So yes, then my trainer dressed me completely. Everything he did, yes.*	9
287. Squeezing toothpaste from tube	*I could not even turn on the shower, or squeeze toothpaste on the toothbrush.*	11
288. Closing buttons	*I could not do up my own buttons with my left.*	13
289. Accelerating on scooter	*So I literally was in pain, as you say 8-9, I just could not accelerate on the motor scooter. At a certain point I was just riding somewhere using my left hand.*	7
290. Opening tap	*And I could not open the faucet either, not cut anything, I have not been able to write for more than 2 years.*	11
291. Washing dishes	*And doing the dishes I could only use one hand and those kinds of things.*	5
292. Closing zipper of bag	*Mostly I let it wait, but I could not even zip up my bag... Closing the zipper of my bag, so I thought, well this is not going right at all.*	13

293.	Lifting heavy objects	*And yes, sometimes yes, it also depends a little on what people want me to do. Sometimes I think well, for example holding a pot that you think, that is all right, but if you have a really full bucket of water, then I leave it to someone else, let's say.*	20
294.	Holding phone	*That I can almost not do. Normally I do it with two fingers, but now with one, or I cannot hold my phone, so that I really noticed, but...*	15
295.	Supporting on wrists when getting out of bed	*Yes, really just leaning on it like this. So when I really was in pain during the gym training, for example when I was getting out of bed, I could not go like this [pushing up with hands].*	16
296.	Turning key		
297.	Riding bicycle		
Adaptations in ADL			
298.	Holding objects differently	*And after that I had an appointment at the physio and he first said, yes you just need to pay attention to... how you grasp everything, so straight and not crosswise.*	8
299.	Cycling with one hand	*So actually for a week I had to go either by car, or I had to ride my bike with one hand.*	13
300.	Typing on phone with one finger	*That I can almost not do. Normally I do it with two fingers, but now with one, or I cannot hold my phone, so that I really noticed, but...*	15
School/study			
Limitations			
301.	Writing	*Well, I have not been able to write for more than two years, that is really impractical if you are a student. With exams and so on also, of course.*	11
		Well, just with writing long stretches and so on, that I do not manage. If, for example, at school I need to write a text, or I need to copy things, then after a few minutes I am done fore.	21
Adaptations			
302.	Typing instead of writing	*Yes, really lifting things, or writing, I do not do that at school any more, for the last six months I have been working on a laptop, too, just because writing I could not do it anymore.*	20
Work			
Limitations			
303.	Holding plates	*Then, too, I could do almost nothing, I could not hold plates. Now I can do it with this hand, with tape I can do it.*	15

Adaptations		
304. Lifting less	*Yes, but then I put the plates down and then I go to the next table. Yes, that is what I do then, but yes. Look, then I think, like, oh well, that is just because the plates are too heavy, next time just three instead of four. Or four instead of five, yes that is how I think, but I never really think, oh that wrist hurts, why not stop.*	15
